# When timing is key: How autocratic and democratic leadership relate to follower trust in emergency contexts

**DOI:** 10.3389/fpsyg.2022.904605

**Published:** 2022-08-03

**Authors:** Florian Rosing, Diana Boer, Claudia Buengeler

**Affiliations:** ^1^Department of Social and Organizational Psychology, Institute of Psychology, University of Koblenz-Landau, Koblenz, Germany; ^2^Department of Human Resource Management and Organization, Institute of Business, Kiel University, Kiel, Germany

**Keywords:** autocratic leadership, democratic leadership, ability, benevolence, trust, interpersonal perception, emergency contexts

## Abstract

In emergency contexts, leaders’ ability to develop others’ trust in them is critical to leadership effectiveness. By integrating functional leadership and team process theories, we argue that democratic and autocratic leadership can create trust in the leader depending on the performance phase of the action team. We further argue that action and transition phases produce different task demands for leadership behavior to enhance trust in the leader, and different leader characteristics (i.e., leader benevolence and leader ability) mediate these effects. The results of a scenario experiment (*N* = 125) and field survey (*N* = 165) among firefighters revealed that autocratic rather than democratic leadership elevates trust in the leader during the action phase by increasing leader ability. In contrast, democratic rather than autocratic leadership enhances trust in the leader during the transition phase by elevating leader benevolence. These findings highlight the importance of leader characteristics in emergencies, demonstrating the value of mixing autocratic and democratic leadership behaviors across different team performance phases to build trust in the leader.

## Introduction

Although follower trust in the leader is a key factor for effective leadership across diverse situations (Dirks and Ferrin, 2002; Burke et al., 2007; Hasel and Grover, 2017), arguably the most critical context for exploring trust is in emergency settings. In this context, followers must trust their leaders and work well together because errors may lead to danger and death (Myers, 2005; Fisher et al., 2010; Kolditz, 2010). If followers do not trust their leader, they may not show strong cohesion and commitment (Weick, 1993; Hamby, 2002).

The previous literature has focused on identifying the behavioral antecedents that increase trust in leaders (Sweeney et al., 2009; Liu et al., 2010; Sweeney, 2010; Kelloway et al., 2012). These studies have implicitly assumed that such antecedents have similar effects on trust in leaders across various tasks and contexts (Lapidot et al., 2007; Colquitt et al., 2011). However, an emerging research strand has suggested that the behavioral antecedents of trust vary in emergency contexts (Lapidot et al., 2007; Sweeney, 2010). Although the current research argues that leadership is critical for mission success and survival (Hällgren et al., 2018), the role of leadership behaviors in this variability remains largely unclear.

We address these research gaps by investigating which leadership behavior functions best in emergency contexts. Many emergency teams perform their work in two recurrent performance phases: action and transition (Kozlowski et al., 1996; Marks et al., 2001; Desmond, 2006, 2008; DeChurch et al., 2011; Farh and Chen, 2018). In action phases (e.g., firefighting), the team acts toward goal accomplishment, whereas in transition phases (e.g., debriefing, reflection, and reexamination), the team focuses on mission analysis, planning, and goal setting. Several studies have suggested that both phases create different team demands, requiring various (functional) leadership behaviors (Marks et al., 2001; Morgeson et al., 2010). In the action phase, proper emergency management is key to ensuring safety, high speed, and efficiency (Yun et al., 2005; Klein et al., 2006; Maynard et al., 2017), as emergencies are unpredictable and demand quick responses (Kozlowski et al., 2009). In the transition phase, demands associated with contemplation and debriefing become salient (Karrasch et al., 2011).

Two “classical” leadership behaviors have been highlighted as significant for effective leadership: autocratic leadership, where solely the leader holds decision-making duties and power, and democratic leadership, where decision-making duties and power are shared with followers (Vroom and Yetton, 1973; Bass, 1990; Van Vugt et al., 2004). While some studies have suggested that autocratic leadership behavior is preferred at the workplace (Isenberg, 1981; Gladstein and Reilly, 1985; Mulder et al., 1986), other studies have favored democratic leadership behavior (Yun et al., 2003, 2005; Sims et al., 2009). Prior work has typically considered non-emergency contexts (Hannah et al., 2009, 2010) and yielded mixed results for the effective of these two leadership behaviors (Berkowitz, 1953; Bass, 1990; Gastil, 1994; Yukl, 2006; Schoel et al., 2011; De Hoogh et al., 2015). As action phases are more risky, urgent, and dangerous than transition phases, we assume that the impact of these two leadership behaviors on follower trust may differ. Thus, the current study investigates how such leadership behaviors are antecedent to follower trust in the leader during both phases.

In addition, to better understand the effectiveness of autocratic and democratic leadership behaviors in emergency contexts, we further examine the mechanisms that underlie the relationship between autocratic or democratic leadership and follower trust during the two phases. Based on functional leadership theory and the integrative model of trust in leadership (Burke et al., 2007), this article investigates the mediating role of leader ability—a key determinant of trust in the leader during action phases (Lapidot et al., 2007)—in the relationship between autocratic leadership and trust in the leader in action phases. Furthermore, we examine the mediating role of leader benevolence—a key determinant of trust in the leader during transition phases (Lapidot et al., 2007)—in the relationship between democratic leadership and trust in the leader in transition phases.

This study extends the literature on leadership in multiple ways. First, we integrate the literature on leadership and team performance phases (Marks et al., 2001) by investigating the impact of autocratic and democratic leadership behavior on follower trust in emergency contexts. Thus, we contribute to the ongoing debate regarding what leadership behaviors are most effective in emergencies (Hannah et al., 2009). This work also offers a nuanced description of the costs and benefits of autocratic and democratic leadership by arguing that their impact on follower trust varies according to the team performance phase. Second, in recognizing key mediators between autocratic and democratic leadership and follower trust, we offer new insights into why these leadership behaviors create trust in emergency contexts. We test our assumptions in the context of firefighting, which possesses many characteristics of organizations operating in emergency contexts (Jankovic et al., 1991; Colquitt et al., 2011; Burtscher et al., 2018). We use a multimethod approach to integrate causal relationships from a scenario experiment (Study 1) with the broader generalizability of an online survey field study (Study 2).

## Background and hypotheses development

### Leadership behaviors and trust in the leader for action teams

Action teams are “teams where members with specialized skills must improvise and coordinate their actions in intense, unpredictable situations” (Edmondson, 2003, p. 1421). Many action teams in emergency contexts accomplish their tasks through dual temporal-phase cycles (McGrath, 1991; Colquitt et al., 2011): action and transition phases. Morgeson et al. (2010) proposed that both team performance phases create different demands, requiring different leadership behaviors. This finding aligns with functional leadership theory (McGrath, 1962), which posits that leadership behaviors are effective when they meet certain functions critical to the team’s needs (Zaccaro et al., 2001). For example, in the action phase, leadership functions include providing resources, monitoring the team, managing team boundaries, and challenging the team (Morgeson et al., 2010). In the transition phase, leaders meet critical team needs through training and developing, providing feedback, engaging in sense-making and sense-giving, defining team missions, and establishing expectations and roles (Morgeson et al., 2010).

This paper focuses on two leadership behaviors, autocratic and democratic, widely used and recommended for risk management in emergency contexts (e.g., Vroom and Yetton, 1973; Gladstein and Reilly, 1985; Yun et al., 2005; Farh and Chen, 2018). We examine the impact of both leadership behaviors on trust in the leader (Hannah et al., 2010), defined as followers’ perception that their leaders are fair and reliable (Rousseau et al., 1998; Dirks, 2000; Schoorman et al., 2007). We suggest that the effect of autocratic and democratic leadership on follower trust varies between the two team performance phases. Specifically, we propose that autocratic leadership enhances trust in the leader during action phases. Vroom and Yetton (1973) emphasized that when the timing is critical, a leader should make decisions alone rather than delegate to team members. Hence, autocratic leaders can make quick, unilateral choices that accelerate decision-making (Eisenhardt, 1989). In particular, novel and unstructured situations require action-oriented leadership (Fiedler, 1964, 1967). Autocratic leadership consolidates administrative control and properly manages resources (Hannah et al., 2009). Thus, we argue that a leader needs to personally take charge of the operation accomplishment to build and maintain follower trust. This initiative accelerates decision-making, reduces uncertainties, and enhances members’ task knowledge (Morgeson, 2005; Kozlowski et al., 2009). We also argue that a democratic leader does not increase follower trust during action phases because these phases enhance the need for direct leader intervention to increase coordination clarity and recover shared understanding (Kozlowski et al., 2009). Therefore, we hypothesize the following:

**H1a**. During the action phase, follower trust in the leader is higher under autocratic than democratic leadership.

We also propose that as the team engages in transition phases, autocratic behaviors decrease in importance, and democratic behaviors become a relevant antecedent for trust in the leader. During transition phases, developmental concerns emerge as demands associated with reflection and reexamination become salient (Kozlowski et al., 2009; Karrasch et al., 2011). Vroom and Yetton (1973) suggested that in situations (1) when time is not essential, (2) the leader alone does not possess the information required to solve a problem, or (3) the interests of leaders and subordinates diverge, democratic leadership is most appropriate for achieving follower trust and consensus-building.

Existing research has demonstrated that followers tend to feel more vulnerable and more willing to scrutinize their leader’s actions and organizational processes after facing autocratic structures during emergencies (Bartunek, 1988; Hurst, 1995; Hannah et al., 2009). Leaders can use democratic behaviors to help followers voice their opinions (Farh and Chen, 2018) and develop their skills and competencies (Sims et al., 2009), which may enhance follower trust. Thus, we argue that a leader must employ democratic leadership to build follower trust during transition phases, enhancing learning opportunities while reducing conflicts among followers and leaders. Accordingly, we hypothesize the following:

**H1b**. During the transition phase, follower trust in the leader is higher under democratic than autocratic leadership.

### The mediating role of trustworthiness in the leader’s ability and benevolence

One mechanism by which autocratic and democratic leadership may exert different effects on trust in the leader is the perception of the leader’s characteristics comprising trustworthiness (Mayer et al., 1995; Cunningham and MacGregor, 2000). Mayer et al.’s (1995) theory distinguished trust from trustworthiness, with three characteristics contributing to the prediction of trust: ability, integrity, and benevolence (Mayer and Davis, 1999; Davis et al., 2000; Gill et al., 2005; Schoorman et al., 2007; Colquitt et al., 2011). This study focuses on leader ability and benevolence, major predictors of follower trust in emergency contexts (Yukl, 2006; Lapidot et al., 2007; Hannah et al., 2009; Sweeney et al., 2009; Sweeney, 2010). We omitted leader integrity from our hypotheses because tests of functional leadership and team process models have produced more meaningful results with regard to the ability and benevolence of leaders than on their integrity (Hannah et al., 2009, 2010; Farh and Chen, 2018). Several studies have found that in situations of heightened vulnerability (i.e., firefighting operations), followers were more attentive to leader behaviors reflecting their ability (compared to leader benevolence; Freitas et al., 2002; Lapidot et al., 2007; Hannah et al., 2009). In such situations, basic security needs are aroused and followers prefer to be with a leader who is determined (Geier, 2016), and competent (Yun et al., 2005; Eberly et al., 2017), partly because in vulnerable situations, followers usually seek to establish a sense of control and mastery (Lazarus, 1966). Conversely, in situations of lowered vulnerability (i.e., debriefing), followers were more sensitive to leader behaviors reflecting benevolence (compared to leader ability; Yun et al., 2005; Lapidot et al., 2007). In such situations, relational needs are aroused and followers prefer to be with a leader who is focused on relationship development (Geier, 2016), and nurturance (Yun et al., 2005; Eberly et al., 2017). Thus, trustworthiness in the leader’s ability was more salient in action phases, and trustworthiness in the leader’s benevolence was more salient in transition phases.

#### The mediating role of leader ability in action phases

We assume that follower trustworthiness in the leader’s ability explains greater trust improvement associated with autocratic leadership compared to democratic leadership during the action phase. Leader ability is “that group of skills, competencies, and characteristics that enable a party to have influence with some specific domain” (Mayer et al., 1995, p. 717). Leader ability means the competence and expertise required to do a specific job with the interpersonal competencies and general wisdom required to succeed in the workplace (Gabarro, 1978; Davis et al., 2000; Burke et al., 2007; Colquitt et al., 2011; Ritzenhöfer et al., 2017).

We suggest that autocratic leadership increases follower perceptions of leader ability by drawing on functional leadership (McGrath, 1962) and team process theories (Marks et al., 2001; Kozlowski et al., 2009). Research on trust suggests that the degree to which the leader takes charge, ensuring a clear direction and an enabling structure, is the degree to which the leader will be perceived as trustworthy in terms of leader ability (Belenky et al., 1985; Hackman, 1992, 2002; Bartone, 2006; Burke et al., 2007). Research on leadership in action teams emphasizes that leaders who provide clear step-by-step directions for followers while enabling adequate resources and structures signal their ability to constructively respond to emergency contexts (Isenberg, 1981; Gladstein and Reilly, 1985; Mulder et al., 1986; Bass, 2008; Martínez-Córcoles, 2018). Therefore, if leaders behave autocratically during action phases, followers are likelier to perceive their leaders as competent than if they behave democratically. In the latter case, followers are less likely to perceive leaders as competent, seeing them as ineffectual (Yun et al., 2005) and indecisive (Gladstein and Reilly, 1985; Mulder et al., 1986; Bass, 1990). Thus, we propose the following hypothesis:

**H2a:** During the action phase, follower perceptions of leader ability are higher under autocratic than democratic leadership.

**FIGURE 1:**
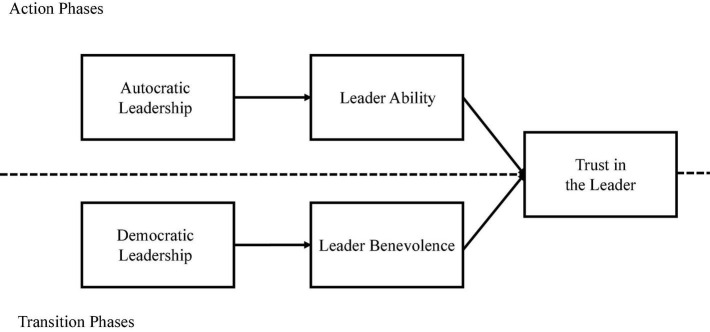
Visual depiction of the proposed research model.

Trustworthiness in the leader’s ability boosts trust in the leader (Mayer et al., 1995; Schoorman et al., 2016; Shao, 2019). Research on trust shows that leader ability enables leaders to maintain their influence within a specific situation (Burke et al., 2007). Given that followers may have little experience, knowledge, and training in some situations, leader ability is a factor that promotes trust (Zand, 1972). Lapidot et al. (2007) argued that when security needs are threatened in emergencies, followers should be more attentive to the leader’s ability to manage the operation and lead the team. Consequently, we predict that autocratic rather than democratic leadership can increase trust in the leader in an action phase through leader ability. Specifically, we expect the leader’s ability to mediate the relationship between autocratic leadership and trust in the leader during action phases (see Figure 1). Hence, we propose the following hypothesis:

**H2b:** In the action phase, follower perceptions of leader ability mediate the positive relationship between autocratic leadership (compared to democratic leadership) and trust in the leader.

#### The mediating role of leader benevolence in transition phases

We assume that follower trustworthiness in the leader’s benevolence explains the greater trust improvement associated with democratic instead of autocratic leadership during the transition phase. Benevolence is a leadership attribute that captures consideration and sensitivity to follower needs and interests (Mayer et al., 1995). Benevolence reflects that leaders are willing to care and want to do good for their followers above what is formally required, even if they do not profit from it (Ritzenhöfer et al., 2017). Thus, we propose that democratic leadership increases follower perceptions of leader benevolence based on functional leadership and team process theories. Research on trust has suggested that the degree to which the leader engages in shared decision-making and provides opportunities to voice opinions increases the perception of leader trustworthiness in terms of benevolence (Hackman, 2002; Burke et al., 2007). Moreover, research on leadership in action teams has suggested that leaders who encourage a high degree of group members’ involvement and participation during decision-making signal that they care about follower welfare and interests during transition phases (Konovsky and Pugh, 1994; Burke et al., 2007; Caldwell and Hayes, 2007). Therefore, if leaders behave democratically in this phase, followers are likelier to perceive the leader as benevolent than if leaders behave autocratically. In the latter case, followers are less likely to perceive their leader as benevolent because they are seen as eroding their sense of control over group decisions (De Cremer, 2007). Hence, we predict the following hypothesis:

**H3a:** During the transition phase, follower perceptions of leader benevolence are higher under democratic than autocratic leadership.

Trustworthiness in the leader’s benevolence boosts trust in the leader (Mayer et al., 1995; Schoorman et al., 2016; Shao, 2019). Mayer et al. (1995) explained that benevolence influences trust because benevolent leaders build a positive attachment to followers and a low motivation to lie. These qualities play a prominent role in building and maintaining trust. Research on action teams shows that in less threatening but stressful situations, such as transition phases, benevolent leaders trigger followers’ needs for nurture and care. In these situations, followers should be more attentive to the leaders’ attributes that reflect their benevolence (Lapidot et al., 2007). Hence, we predict that democratic rather than autocratic leadership can increase follower trust during transition phases by enhancing perceptions of leader benevolence. Specifically, we expect leader benevolence to mediate the relationship between democratic leadership and trust in the leader. We propose the following hypothesis:

**H3b:** In the transition phase, follower perceptions of leader benevolence mediate the positive relationship between democratic leadership (compared to autocratic leadership) and trust in the leader.

### Overview of the current work

Following recommendations for confirming our results’ robustness and generalizability (Chen et al., 2021), we used a multimethod approach to examine our hypotheses. We conducted two studies among professional firefighters. In Study 1, we conducted a scenario experiment to test our hypotheses in a controlled research context (Aguinis and Bradley, 2014). We then conducted a cross-sectional field study to enhance external validity. In Study 2, we replicated and extended the findings from Study 1 using cross-sectional data from a sample of 165 firefighters to test our hypotheses in an emergency context.

## Study 1

### Method

#### Participants

In this study, 125 firefighters participated, of which 89% were male, ages 16 to 62 years (*M* = 32.35, *SD* = 9.99), with an average of 16 years of work experience. Firefighters were recruited via professional firefighting conferences, department visits, email news briefs, blogs, and forums. All firefighters volunteered to complete the study.

#### Design and experimental procedure

This study used a 2 × 2 between-subject design, with the leader’s behavior (autocratic/democratic) as the first factor and the temporal phase (action/transition phase) as the second factor. Participants were randomly assigned to one of four vignettes [autocratic/action phase (*n* = 31), democratic/action phase (*n* = 32), autocratic/transition phase (*n* = 30), democratic/transition phase (*n* = 32)] to control for order effects.

The online experiment included a screening page, informed consent, instructions, the vignette (to manipulate the type of leadership behavior and temporal phase), and a questionnaire (Geier, 2016). The questionnaire included the dependent measures and controls. We informed participants that they were participating in a study that “examines critical success factors of effective teamwork” and that they were going to read a scenario and would respond to questions about that scenario. Specifically, they were asked to imagine that they were experiencing the written scenario and assume the role of a fire brigade member.

#### Vignette development

We began vignette development by constructing a set of draft scenarios based on nine in-depth interviews in which firefighters were asked about realistic examples of leadership behaviors during the action and transition phases (Ashill and Yavas, 2006; Levy, 2006; Aguinis and Bradley, 2014; Rosing et al., 2022; see Appendix 1). The most frequently mentioned examples of autocratic and democratic leadership within action and transition phases were selected to create the vignettes (Leicher and Mulder, 2016). The vignettes were reviewed by three researchers (who were not part of the research team) and two firefighters until consensus was achieved on their true-to-life correctness (Hughes and Huby, 2002; McAlearney, 2006; see Table 1).

**TABLE 1:** Vignettes used in Study 1.

Action phase		Transition phase	
Autocratic leadership	Democratic leadership	Autocratic leadership	Democratic leadership
We were sitting together with the emergency services at 6 a.m., shortly before closing time, when the gong rang, and a voice shouted from the loudspeaker: “Hauptstraße 14 (City), building fire with human lives in danger!” On-site, we received the following message from our chief via radio: “To the emergency teams on the approach to Hauptstraße: Building fire of a restaurant, presumably fat explosion. Fire glow visible from the roof truss. Four people injured and in danger of their lives! Nobody left in the building.” Furthermore, our head of operations ordered, “Immediate emergency care of all acutely injured persons! Establish and secure vital functions! Afterward, establish transportability as last practiced. I expect these measures to be implemented immediately!”	We were sitting together with the emergency services at 6 a.m., shortly before closing time, when the gong rang and a voice shouted from the loudspeaker: “Hauptstraße 14 (City), building fire with human lives in danger! On-site, we received the following message from our chief via radio: “To the emergency teams on the approach to Hauptstraße: Building fire of a restaurant, presumably fat explosion. Fire glow visible from the roof truss. Four people injured and in danger of their lives! Nobody left in the building.” The fire chief asked all forces, “Do you want to fight the fire first?” One comrade answered, “I’d first get the injured ready for transport.” “That’s a good idea,” the chief replies.	Following a large, stressful fire operation, a transition phase was held with all the emergency services involved. One comrade said, “We have been on duty for 30 h, and the rest period for the next duty cannot be kept. I have to relieve my wife, who is looking after our child, and two comrades have been injured. Under these circumstances, it would be good to adjust the duty roster.” Our chief ordered, “The duty roster must be maintained in this way! I expect all forces to adhere to it!” One comrade said, “I think it would be better to keep the duty roster…” “No, no, no, no, no, no,” the chief interrupted.	Following a large, stressful fire operation, a transition phase was held with all the emergency services involved. One comrade said, “We have been on duty for 30 h, and the rest period for the next duty cannot be kept. I have to relieve my wife, who is looking after our child, and two comrades have been injured. Under these circumstances, it would be good to adjust the duty roster.” Our chief asked all units, “What do you say, shall we change the duty roster? We have enough troops in reserve.” A comrade said, “Yes, under the circumstances, that would be a good solution.” “Yes, good idea, then you’ll be rested and ready for the next service,” the chief replied.

### Measures

Unless otherwise stated, the measures in this study used a 7-point Likert scale (1 = strongly disagree to 7 = strongly agree).

#### Autocratic leadership behavior

We measured the effectiveness of manipulating autocratic leadership with six items from the autocratic leader behavior scale (De Hoogh et al., 2004). The Cronbach’s alpha score was 0.92. Examples included items such as “the leader makes decisions in an autocratic way” and “the leader often pushes his/her opinions.”

#### Democratic leadership behavior

We measured the effectiveness of manipulating democratic leadership using five items from the participative decision-making questionnaire (Arnold et al., 2000). Sample items included “the leader encourages group members to express ideas/suggestions” and “the leader gives all group members a chance to voice their opinions.” The Cronbach’s alpha score was 0.70.

#### Leader ability and benevolence

Mayer and Davis (1999) developed a measure of trustworthiness that included subscales of leader ability and benevolence. We used six items to measure leader ability (“the leader is very capable of performing his job,” α = 0.96) and five items to study leader benevolence (“the leader is very concerned about followers’ welfare,” α = 0.97).

#### Trust in the leader

We used a three-item scale developed by Giessner and van Knippenberg (2008) to assess overall trust in the leader. The items were as follows: “I trust the leader absolutely,” “I think this leader does the right things,” and “I think this leader is trustworthy” (α = 0.95).

#### Control variables

We controlled for firefighters’ age (continuously in years), gender (1 = male, 2 = female), and job tenure (continuously in years). The results remained unchanged with or without these controls in the model.

#### Data analysis

We analyzed our data in several steps. First, the variance inflation factor (VIF) was calculated for each variable to determine multicollinearity (Suen, 1990). Second, a one-way analysis of variance (ANOVA) tested the effects of leadership behaviors on follower trust (*H1a-b*) and perceptions of the leader’s abilities and benevolence (*H2a* and *H3a*). We also used planned comparison (Rosenthal and Rosnow, 1985) tests to compare the means between the conditions. Third, to analyze H2b and H3b, we used the PROCESS tool in SPSS (Hayes, 2012). We chose model 4 of this tool with 5,000 bootstraps and, as recommended (Hayes and Preacher, 2014), a confidence interval of 95% for estimating the respective effects.

### Results

#### Multicollinearity

We assessed the absence of multicollinearity with VIF scores of less than 10 (VIF < 10 = no serious multicollinearity; Cohen et al., 2003; Neubert and Taggar, 2004). The results of our multicollinearity analysis showed that the VIF scores ranged within acceptable values from 1.09 to 2.87 (VIF = 2.45 for leader ability, 2.52 for leader benevolence, 2.82 for age, 1.09 for gender, and 2.87 for job tenure), indicating an extremely low level of multicollinearity in our study (Dingel and Wei, 2014).

#### Manipulation checks

Table 2 presents descriptive statistics and intercorrelations among all study variables. Table 3 shows the means and standard deviations for all experimental conditions. We analyzed the manipulation checks in two steps. First, we analyzed whether our leadership manipulation in the action phase was effective. The results of a univariate ANOVA indicated that participants assigned the autocratic leadership condition perceived the leader as being more autocratic than participants assigned the democratic condition [*M* = 5.10, *SD* = 1.18 vs. *M* = 2.39, *SD* = 1.52, with *t*(121) = 8.95, *p* < 0.01]. In contrast, participants assigned the democratic leadership condition perceived the leader as more democratic than participants assigned the autocratic condition [*M* = 2.76, *SD* = 0.90 vs. *M* = 4.97, *SD* = 0.94, with *t*(121) = −10.59, *p* < 0.01].

**TABLE 2:** Descriptive statistics and intercorrelations among Study 1 variables.

Variable	*M*	*SD*	1	2	3	4	5	6	7
1. Age	32.35	9.99	–						
2. Gender^a^	1.11	0.32	−0.23*	–					
3. Job tenure	15.76	9.83	0.80**	−0.29**	–				
4. Autocratic leadership	4.30	1.90	0.01	0.02	0.03	–			
5. Democratic leadership	3.87	1.57	–0.03	0.10	–0.05	−0.60**	–		
6. Leader ability	3.85	1.76	–0.16	0.05	–0.16	0.06	0.45**	–	
7. Leader benevolence	3.99	1.96	−0.21*	0.00	–0.17	−0.38**	0.69**	0.77**	–
8. Trust in the leader	3.88	1.93	−0.22*	0.01	–0.17	0.02	0.44**	0.90**	0.78**

N = 125; ^a^1 = male; 2 = female; *p < 0.05, **p < 0.01.

**TABLE 3:** Mean values, standard deviations, and significances of differences between experimental conditions (Study 1).

	Leadership Behavior		
Variable	Autocratic *M* (*SD*)	Democratic *M* (*SD*)	*t (df)*
***Action phase***			
MC: Autocratic leadership	5.10 (1.18)	2.39 (1.52)	8.95** (121)
MC: Democratic leadership	2.76 (0.90)	4.97 (0.94)	–10.59** (121)
Leader ability	4.21 (1.41)	3.29 (1.76)	2.54* (121)
Trust in the leader	4.22 (1.67)	3.22 (1.90)	2.58* (121)
***Transition phase***			
MC: Autocratic leadership	6.30 (0.54)	3.56 (1.32)	8.95** (121)
MC: Democratic leadership	2.27 (0.87)	5.35 (0.55)	–14.66** (121)
Leader benevolence	1.66 (1.01)	5.84 (0.98)	–13.09** (121)
Trust in the leader	2.41 (1.31)	5.58 (1.14)	–8.13** (121)

N = 125; *p < 0.05, **p < 0.01.

Second, we used a univariate ANOVA to test whether our manipulation of leadership in the transition phase was successful. The results again showed that participants assigned the autocratic leadership condition perceived the leader as being more autocratic than participants assigned the democratic condition *[M* = 6.30, *SD* = 0.54 vs. *M* = 3.56, *SD* = 1.32, with *t*(121) = 8.95, *p* < 0.01]. In contrast, participants assigned the democratic leadership condition perceived the leader as being more democratic than participants assigned the autocratic condition [*M* = 5.35, *SD* = 0.55 vs. *M* = 2.27, *SD* = 0.87, with *t*(121) = −14.66, *p* < 0.01]. In conclusion, all manipulations worked as expected.

#### Hypotheses testing

**FIGURE 2:**
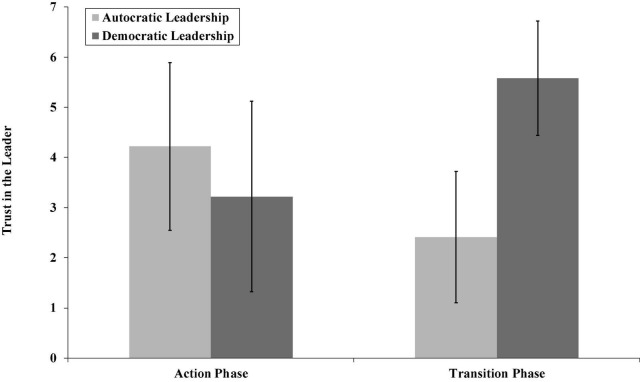
The relationship between autocratic and democratic leadership and trust in the leader for the action and transition phase (Study 1).

**FIGURE 3:**
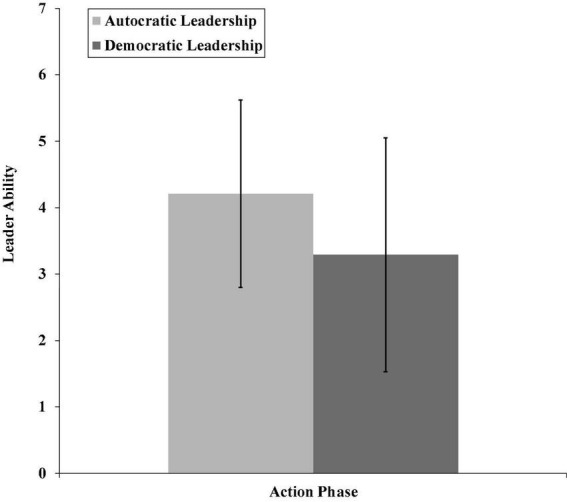
The relationship between autocratic and democratic leadership and leader ability for the action phase (Study 1).

We supported H1a since performing autocratic leadership behavior during the action phase resulted in higher trust ratings (*M* = 4.22, *SD* = 1.67) than performing democratic leadership (*M* = 3.22, *SD* = 1.90; *p* < 0.05). We also supported H1b since performing democratic behavior during the transition phase resulted in higher trust in the leader (*M* = 5.58, *SD* = 1.14) than performing autocratic leadership (*M* = 2.41, *SD* = 1.31; *p* < 0.01; see Figure 2). Moreover, we supported H2a since autocratic leadership resulted in higher leader ability ratings (*M* = 4.21, *SD* = 1.41) during the action phase than democratic leadership (*M* = 3.29, *SD* = 1.76; *p* < 0.05, see Figure 3) and also supported H2b since the indirect effect of autocratic leadership (as compared with democratic leadership) on follower trust during the action phase through leader ability was significant (effect = –0.90, 95% CI[–1.63, –0.13]).

**FIGURE 4:**
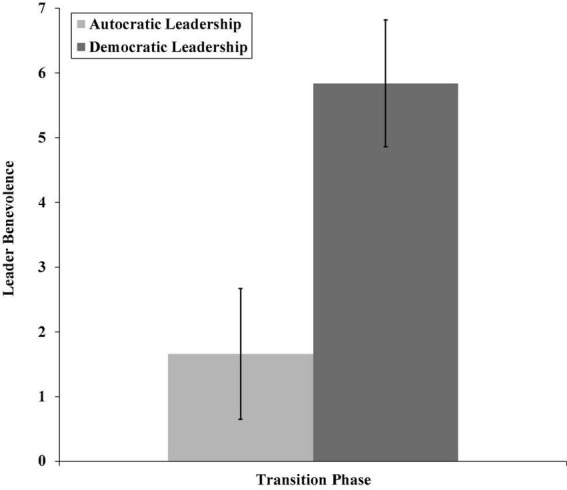
The relationship between autocratic and democratic leadership and leader benevolence for the transition phase (Study 1).

Finally, we supported H3a since leader benevolence ratings were higher for democratic leadership behavior (*M* = 5.84, *SD* = 0.98) during the transition phase than for autocratic leadership behavior (*M* = 1.66, *SD* = 1.01; *p* < 0.01, see Figure 4). Furthermore, in the transition phase, the indirect effect of democratic (rather than autocratic) leadership behavior on follower trust through leader benevolence was significant (effect = 3.95, 95% CI[2.84, 5.57]), supporting H3b.

### Discussion

Using a scenario-based experimental methodology, we designed Study 1 to provide an experimental test of the impact that autocratic and democratic leadership has on follower trust. Study 1 supported that the influence of autocratic and democratic leadership on follower trust varies depending on the team performance phase since the action and transition phases involve different task demands and thus leadership requirements. We found that autocratic rather than democratic leadership elevates follower trust by increasing leader ability in the action phase. In contrast, democratic rather than autocratic leadership enhances follower trust during transition phases by elevating leader benevolence. Our scenario-based experimental methodology had the advantage of isolating the effects of autocratic and democratic leadership (Antonakis et al., 2010). However, the drawback was that respondents could not directly experience the team performance phase to which they were responding (Belschak and Den Hartog, 2009). Therefore, we conducted a second study in the context of firefighting to replicate and extend the results from Study 1 using a different method involving a sample of firefighters who actually experience leader behaviors in extreme operations.

## Study 2

### Method

#### Sample, design, and procedure

We recruited firefighters through professional firefighting conferences, department visits, email news briefs, blogs, and forums to participate in an online survey. Various recruitment methods restricted us from calculating an exact response rate. However, of the 576 firefighters registered for participation, 165 (29%) completed the survey. All participants worked for firefighting departments in Germany. The study sample consisted of 147 males and 18 females, averaging 36 years of age (*SD* = 10.77) and 18 years of work experience (*SD* = 10.16). All firefighters volunteered to complete the study.

We used the critical incident technique (Aquino et al., 2006; Wang et al., 2018) to elicit salient experiences of action and transition phases. First, we asked participants to complete the following task: to think back over the last months as a firefighter and recall the last incident where they experienced an acute, serious, and dangerous firefighting operation. After thinking about this incident, respondents answered questions about leadership behaviors and trust in the leader. Second, we asked participants to complete another task: to think back over the last month as a firefighter and recall the last incident where they experienced a transition phase, such as a debriefing. Again, after deliberating about this critical incident, participants answered the questions about leadership behaviors and trust in the leader.

### Measures

Unless otherwise stated, we used measures on a 7-point Likert scale (1 = strongly disagree to 7 = strongly agree).

#### Autocratic leadership behavior

As in Study 1, we measured autocratic leadership using the six-item autocratic leader behavior scale developed by De Hoogh et al. (2004). The Cronbach’s alpha score was 0.75 for the action phase and 0.87 for the transition phase.

#### Democratic leadership behavior

We assessed democratic leadership using five items from the participative decision-making questionnaire, as in Study 1 (Arnold et al., 2000). The Cronbach’s alpha score was 0.89 for the action phase and 0.94 for the transition phase.

#### Leader ability and benevolence

As in Study 1, we used a measure developed by Mayer and Davis (1999) to assess leader ability and benevolence. We used six items to measure leader ability (α = 0.96 for action phase, α = 0.97 for transition phase) and five items for leader benevolence (α = 0.90 for the action phase, α = 0.95 for the transition phase).

#### Trust in the leader

Follower trust was measured using the three-item scale developed by Giessner and van Knippenberg (2008), as in Study 1. The Cronbach’s alpha score was 0.93 for the action phase and 0.96 for the transition phase.

#### Control variables

As in Study 1, we controlled for firefighter age, gender, and job tenure. The results remained unchanged with or without these controls in the model.

#### Data analysis

We analyzed our data using ordinary least square regression to check for H1a, H1b, H2a, and H3a and examined the statistical significance of the difference between the two means by investigating whether the two 95% confidence intervals overlapped (Schenker and Gentleman, 2001; Ryu and Cheong, 2017). Moreover, we used bootstrapping and bias-corrected confidence intervals (95%) to analyze H2b and H3b. All mediation analyses were performed with the PROCESS tool in SPSS (Hayes, 2012). Analyses were repeated without control variables, resulting in findings similar to those reported here.

### Results

#### Multicollinearity

The findings of our multicollinearity analysis showed that the VIF values ranged within acceptable values, from 1.11 to 2.76 in the action phase (VIF = 1.74 for autocratic leadership, VIF = 1.07 for democratic leadership, VIF = 2.76 for leader ability, 2.43 for leader benevolence, 2.47 for age, 1.11 for gender, and 2.37 for job tenure) and from 1.02 to 3.61 in the transition phase (VIF = 1.02 for autocratic leadership, VIF = 1.96 for democratic leadership, VIF = 3.61 for leader ability, 3.40 for leader benevolence, 2.44 for age, 1.10 for gender, and 2.34 for job tenure). Thus, no severe multicollinearity problems were present in our research model.

#### Hypothesis testing

Tables 4, 5 provide the means, standard deviations, and correlation coefficients among the study variables for both temporal phases. Table 6 presents the findings of the regression analysis. First, the results of the regression analysis showed that the effect of autocratic leadership on trust in the leader was significantly positive (*b* = 0.75, *SE* = 0.09, *p* < 0.01, 95% CI[0.56, 0.91]), and the effect of democratic leadership was also significantly positive (*b* = 0.14, *SE* = 0.06, *p* < 0.05, 95% CI[0.18, 0.25]) in the action phase. Supporting H1a, the 95% confidence intervals of the two estimates did not overlap, hence the two were statistically significantly different from one another.

**TABLE 4:** Descriptive statistics and correlations for the action phase (Study 2).

Variable	*M*	*SD*	1	2	3	4	5	6	7	8
1. Age	35.62	10.78	–							
2. Gender^a^	1.11	0.31	−0.31**	–						
3. Job tenure	18.45	10.16	0.75**	−0.20**	–					
4. Autocratic leadership	5.54	1.01	–0.02	0.05	0.07	–				
5. Democratic leadership	2.97	1.56	0.04	–0.06	0.02	–0.01	–			
6. Leader ability	5.87	1.23	–0.10	0.06	–0.08	0.62**	0.15	−		
7. Leader benevolence	5.72	1.19	–0.09	0.08	–0.05	0.56**	0.17*	0.75**	–	
8. Trust in the leader	5.75	1.39	–0.11	0.03	–0.10	0.54**	0.15	0.81**	0.72**	–

N = 165; ^a^1 = male, 2 = female; *p < 0.05, **p < 0.01.

**TABLE 5:** Descriptive statistics and correlations for the transition phase (Study 2).

Variable	*M*	*SD*	1	2	3	4	5	6	7	8
1. Age	35.62	10.78	–							
2. Gender^a^	1.11	0.31	−0.31**	–						
3. Job tenure	18.45	10.16	0.75**	−0.02**	–					
4. Autocratic leadership	4.15	1.48	0.02	0.05	0.03	–				
5. Democratic leadership	5.50	1.58	0.02	0.05	0.01	0.07	–			
6. Leader ability	5.82	1.33	–0.02	0.03	–0.03	0.10	0.67**	–		
7. Leader benevolence	5.89	1.29	–0.04	0.05	–0.03	0.06	0.64**	0.83**	–	
8. Trust in the leader	5.73	1.47	–0.05	0.07	–0.04	0.05	0.62**	0.84**	0.80**	–

N = 165; ^a^1 = male, 2 = female; *p < 0.05, **p < 0.01.

**TABLE 6:** Effects of leadership behaviors on trust in the leader and leader ability and benevolence (Study 2).

	Action phase				Transition phase			
	Leader ability		Trust in the leader		Leader benevolence		Trust in the leader	
	*b*	*SE*	*b*	*SE*	*b*	*SE*	*b*	*SE*
Age	0.00	0.01	–0.00	0.01	–0.00	0.01	–0.01	0.01
Gender^a^	0.11	0.26	–0.04	0.30	–0.11	0.29	–0.01	0.32
Job tenure	–0.02	0.01	–0.02	0.01	–0.00	0.01	0.00	0.01
Autocratic leadership	0.76**	0.07	0.75**	0.09	0.00	0.06	0.01	0.06
Democratic leadership	0.13*	0.05	0.14*	0.06	0.53**	0.05	0.58**	0.06
*R* ^2^	0.42		0.33		0.42		0.39	
Adjusted *R*^2^	0.40		0.31		0.39		0.37	

N = 165; ^a^1 = male; 2 = female; *p < 0.05, **p < 0.01; unstandardized coefficients are reported.

Second, the effect of democratic leadership on trust in the leader was significantly positive (*b* = 0.58, *SE* = 0.06, *p* < 0.01, 95% CI[0.46, 0.69]), and the effect of autocratic leadership was non-significant in the transition phase (*b* = 0.01, *SE* = 0.06, *p* > 0.05, 95% CI[–0.11, 0.13]). Supporting H1b, the 95% confidence intervals of the two estimates did not overlap. Thus, we concluded that they were different from each other.

Third, there was a positive significant effect of autocratic leadership on leader ability in the action phase (*b* = 0.76, *SE* = 0.07, *p* < 0.01, 95% CI[0.60, 0.89]). There was also a positive significant effect for democratic leadership (*b* = 0.13, *SE* = 0.05, *p* < .05, 95% CI[0.03, 0.29]). Supporting H2a, the 95% confidence intervals of the two estimates were significantly different from each other as the confidence intervals of the two estimates did not overlap.

Fourth, the findings also supported H2b since the indirect effect on trust in the leader through leader ability in the action phase was significantly positive for autocratic leadership (effect = 0.65, 95% CI[0.44, 0.89]) and insignificant for democratic leadership (effect = 0.11, 95% CI[–0.01, 0.23]). Fifth, there was a significant positive effect of democratic leadership on leader benevolence in the transition phase (*b* = 0.53, *SE* = 0.05, *p* < 0.01, 95% CI[0.42, 0.62]) and an insignificant effect for autocratic leadership (*b* = 0.00, *SE* = 0.06, *p* > 0.05, 95% CI[–0.11, 0.11]). Thus, the results supported H3a since the 95% confidence intervals of the two estimates did not overlap, hence the two estimates were statistically significantly different from one another. Sixth, our results also supported H3b, as the indirect effect on follower trust through leader benevolence in the transition phase was significantly positive for democratic leadership (effect = 0.41, 95% CI[0.25, 0.61]) but insignificant for autocratic leadership (effect = 0.05, 95% CI[–0.10, 0.20]).

### Discussion

Study 2 replicated and extended the results from Study 1 using a different research method. In line with findings obtained in Study 1, Study 2 supported that autocratic leadership, compared to democratic leadership, elevates follower trust in the leader by increasing leader ability in the action phase. In addition, we demonstrated that democratic compared to autocratic leadership enhances follower trust in the leader during the transition phase by elevating leader benevolence.

## General discussion

Our findings demonstrate that the influence of autocratic and democratic leadership on follower trust differs between action and transition phases. We find that follower trust is more strongly related to autocratic rather than democratic leadership during the action phase, whereas follower trust during the transition phase is more strongly related to democratic rather than autocratic leadership. Moreover, our results show that autocratic leaders have higher abilities than democratic leaders in action phases, whereas democratic leaders are more benevolent than autocratic leaders in transition phases. Furthermore, we find that the link between autocratic leadership and trust is mediated by leader ability in action phases, whereas the link between democratic leadership and trust is mediated by leader benevolence in transition phases.

### Theoretical implications

This article contributes to the leadership and trust literature by demonstrating the necessity of considering situational factors when assessing the effects of autocratic and democratic leadership on follower trust. In line with other studies on follower trust (e.g., Dirks, 2000; Burke et al., 2007) and autocratic and democratic leadership (Lewin and Lippitt, 1938; Gastil, 1994; Foels et al., 2000; Schoel et al., 2011), we demonstrate that explicit consideration of the context provides a better description of the effects of autocratic and democratic leadership on follower trust.

Departing from prior research that has mainly considered these leadership behaviors in isolation (e.g., Mulder et al., 1971; Gladstein and Reilly, 1985; Eisenhardt, 1989; Bass, 1990; Bass and Riggio, 2006; Sweeney et al., 2009), we directly compare autocratic and democratic leadership behaviors to clarify the conflicting findings in the literature on the effectiveness of both behaviors for instilling follower trust. Thus, we provide insights into when and why autocratic and democratic leadership behaviors foster follower trust in emergency contexts and contribute to the debate regarding the limits and benefits of both behaviors (Berkowitz, 1953; Bass, 1990; Gastil, 1994; Yukl, 2006; Schoel et al., 2011; De Hoogh et al., 2015).

Conceptually, we extend the leadership and trust literature by considering the critical role of team process phases (Marks et al., 2001), showing that the influence of autocratic and democratic leadership on follower trust manifests through different task demands encountered in the action and transition phases. This finding is consistent with functional leadership (McGrath, 1962) and team process theory (Kozlowski et al., 2009; Morgeson et al., 2010), suggesting that action and transition phases produce different task demands for leadership behavior. By demonstrating that the influence of autocratic and democratic leadership on follower trust differs significantly across the two phases, we highlight an important temporal condition that may help explain some of the inconsistencies in previous research regarding the effectiveness of autocratic versus democratic leadership at the workplace (Schoel et al., 2011), proposing its impact to both positively and negatively affect the working environment.

Previous research on the effectiveness of autocratic leadership has highlighted positive and negative consequences (e.g., Bass, 1990; Gastil, 1994; Foels et al., 2000; De Hoogh et al., 2015). This study supports that autocratic leadership is not always costly and sometimes fosters follower trust. Specifically, autocratic leadership during action phases promotes follower trust and perceptions of the leader’s abilities. This finding aligns with normative models (Vroom and Yetton, 1973; Vroom and Jago, 1978), suggesting that autocratic leadership allows for fast decision-making processes and facilitates reaction times in time-sensitive situations. Moreover, this study informs leadership research by answering the call for more research on different behaviors of leadership in emergency contexts (Hannah et al., 2009; Hannah and Parry, 2014). In particular, this study shows that autocratic leadership can have functional value for follower trust situations of heightened vulnerability.

Previous studies have largely focused on the positive effects of democratic leadership on team performance and effectiveness in the workplace (Kushell and Newton, 1986; Foels et al., 2000). Scholars have suggested that followers do not prefer domineering leadership behaviors but are more efficient and satisfied when they participate in decision-making (Gastil, 1994). Our results show a functional value for democratic leadership during transition phases and a dysfunctional value during action phases. In particular, we demonstrate that democratic leadership fosters follower perceptions of leader benevolence and trust in the transition phase. In contrast, democratic leadership is unrelated to follower perceptions of the leader’s abilities in action phases.

These findings align with the normative model (Vroom and Yetton, 1973; Vroom and Jago, 1978) and social exchange processes (Foels et al., 2000; Bass, 2008), suggesting that democratic leadership may operate by establishing care and consideration for followers in situations where time is not essential and where the leader does not have the information required to solve the problem alone (Yukl, 2006). Thus, our study informs the debate on leadership effectiveness, demonstrating a functional and dysfunctional value for participatory and decentralized leadership behaviors in the two team performance phases.

### Limitations and future research

This research has certain limitations, highlighting possible directions for future study. First, further research is needed concerning the operation of trust for emergency jobs with recurring team performance phases. Future work can thus expand this study and focus on how trust in the leader develops throughout the performance phase (Sweeney, 2010). Future studies could include questions and measures to allow researchers to examine whether trust in the leader developed during a transition phase might transfer to an action phase. For example, Hannah et al. (2009) suggested that appropriate leadership behavior before an emergency event may allow leaders to be more autocratic during the emergency event based on the trust they have already built.

Second, we limit the analyzed leadership types to the two behaviors identified by Lewin and Lippitt (1938): autocratic and democratic. In emergency contexts, the development and operation of trust may include other leadership behaviors. However, research suggests that adaptive and flexible leadership should consider a variety of leadership types, such as transformational and transactional leadership (e.g., Arnold et al., 2016; Geier, 2016; Eberly et al., 2017) and shared leadership (e.g., Klein et al., 2006; Ramthun and Matkin, 2014).

Third, our study narrowly defines autocratic and democratic leadership as two variants of decision-making (Vroom and Yetton, 1973; Vroom and Jago, 1988). We describe autocratic leadership as forbidding subordinate involvement in decisions and democratic leadership as group decision-making. However, future research might differentiate between other variants of decision-making, such as consultation.

Fourth, we investigate only two leader characteristics comprising trustworthiness (ability and benevolence) that serve as mechanisms to explain the impact of autocratic and democratic leadership on trust. Previous research has suggested that future research should explore other mechanisms (Mayer et al., 1995; Dirks, 2000; Mayer and Gavin, 2005). Thus, we encourage future research to examine other underlying mechanisms or moderators on the development and operation of follower trust, such as leader integrity, individual propensity to trust, and individual perceived risks (Burke et al., 2007).

Fifth, a significant limitation of this study is our inability to test causality within our research design, particularly as our mediators and dependent variables are measured through cross-sectional self-reports. Future longitudinal studies and diary studies should address this issue.

Sixth, we find that trustworthiness in the leader’s ability and trustworthiness in the leader’s benevolence highly correlate with trust in the leader, raising concerns about content overlap. Therefore, we use well-validated scales to focus on nonoverlapping constructs (Colquitt and Rodell, 2011). We also measure and account for multicollinearity in our analyses. Our findings support the measurement of our constructs, and the low likelihood that construct content overlap is a concern in the present research. Future studies should account for overlapping content correlation in trust research.

### Practical implications

Our findings show that functional and dysfunctional values exist for autocratic and democratic leadership concerning follower trust. These findings inform the debate on whether autocratic and democratic leadership are important leadership tools in emergency contexts (e.g., Hannah et al., 2010). Our findings highlight that it is important for leaders to understand the positive impact they can have on follower trust by enacting a mix of autocratic and democratic leadership behaviors across performance phases. Furthermore, leaders should be aware of the dynamic task features of emergency contexts and adjust their leadership behaviors depending on the phase to which they are exposed.

We also show advantages to employing both leadership behaviors and providing a framework for leaders to follow, depending on the team performance phases (Marks et al., 2001). For teams facing action phases (e.g., fire missions, surgeries), autocratic leadership is the most appropriate, as units must be able to immediately operate at peak performance and full speed. The team cannot afford to slow down the treatment process for the participation required in democratic teams (Yun et al., 2005; Lorinkova et al., 2013). In contrast, when units are exposed to transition phases (e.g., operational debriefings), democratic leadership is the most appropriate choice, facilitating learning opportunities, feelings of identity, and commitment of the units.

Leadership development activities can also help raise leaders’ awareness regarding how their behaviors may or may not lead to follower trust, depending on the leader’s abilities and benevolence. Leaders can then learn to adjust their behavior as required (De Hoogh et al., 2015). For example, leaders can use autocratic leadership techniques for action-related events.

### Conclusion

This study provides meaningful insights into the relative benefits of autocratic and democratic leadership. Previous research has not compared autocratic and democratic leadership in emergency contexts, and the unique impact of both leadership behaviors on follower trust remains unknown. Our findings suggest that it is important for leaders to understand the positive impact they can have on follower trust by enacting a mix of autocratic and democratic leadership across different team performance phases.

## Data availability statement

The raw data supporting the conclusions of this article will be made available by the authors, without undue reservation.

## Ethics statement

The studies involving human participants were reviewed and approved by University of Koblenz-Landau. The patients/participants provided their written informed consent to participate in this study.

## Author contributions

All authors contributed to the design and implementation of the research, to the analysis of the results, to the writing of the manuscript, and provided final approval of the version to be published.

## Appendix 1: Pilot study

To construct a set of draft vignettes of autocratic and democratic leadership in emergency contexts and ensure their validity, we first conducted a qualitative pilot study using an in-depth interview technique with a firefighter sample (Belkin and Rothman, 2017).

### Method

#### Participants

Nine firefighters participated in the study: all males aged 24 to 40 years old (*M* = 26 years, *SD* = 5.94), averaging 11 years of work (*SD* = 5.87). The participants were employed across four departments in the midwestern region of Germany. The sample included two lieutenants, one driver/ladder operator, and six firefighters.

#### Procedure

A trained, professional interviewer collected data during face-to-face interviews of approximately 90 min. We used a semi-structured interview guide with open-ended questions (Taylor and Bogdan, 1998), and all tape-recorded interviews were transcribed verbatim for the data analysis. Participants were recruited using a snowball sampling technique (McAlearney, 2006). The original sample of firefighters was generated by academic staff and extended by informants asked to suggest additional experts. Data saturation was judged to have occurred if interviews offered repetitive information and ideas and no new information emerged (Strauss and Corbin, 1990; Morse, 2000).

Firefighters were free to talk about personal thoughts and feelings and explore real-life situations (Miles and Huberman, 1994; Ashill and Yavas, 2006; Levy, 2006). They were asked questions such as, “What do you think you need from your leader during action phases? And during transition phases? Are some leadership behaviors particularly essential in each phase?” They were then asked to narrate critical leadership behavior incidents in their daily work experiences during and following various operations. They were also questioned about their preferences and expectations regarding the leader’s behavior and its relationship to trust development and erosion.

#### Data analysis

Our analysis followed the inductive analysis technique by searching for patterns and identifying data themes (Strauss and Corbin, 1990; Haddon et al., 2015; Pratt et al., 2019). Two independent researchers read the interview transcripts several times, identifying themes, key ideas, and recurring statements shared by interviewees (Miles and Huberman, 1994). Coding was descriptive and open and remained close to the firefighters’ language (Strauss and Corbin, 1990).

### Results

In this section, the results are summarized into two distinct themes. The first theme answered the question, “What do you think you need from your leader during action phases?” The second theme concerned the forms of leadership displayed in the transition phase. Verbatim quotations were chosen as representative of the qualitative data. Below, we discuss each theme in more detail.

#### Theme 1: Leadership in the action phase

The most critical leadership behavior in the action phase was the leaders’ ability to be decisive and set directions. The importance of leaders telling followers what to do while effectively inhibiting discussions was considered important to provide the opportunity to act quickly and follow an operation plan. This attitude was evidenced by the representative quotes below:

Leadership should set direction. It goes like this. Otherwise, it wastes time and delays the whole process. (Participant 1) When the order is given, the order is given. That’s what you do. Orders create silence. It’s a very clear job. There’s nothing to discuss what you have to do. […] It’s not up for discussion. Outside it’s hectic and chaotic, but simple commands make you calm in a situation. […] Clear simple orders are required. (Participant 2) Discussions are not at all beneficial and protract the mission. This makes it more tense between the forces. […] In the end, I do something wrong. (Participant 7) You should never reject a command. It gets unpredictable at this moment. (Participant 4)

Establishing a sense of hierarchy was emphasized, helping to bundle, process, and analyze all incoming information. The leader was seen as a contact person whom they could turn to in case of need, as evidenced by the quotes below:

One cannot act democratically in such a situation. Gotta maintain hierarchy; can’t have a great debate there. I’m just an underling. (Participant 8) We have a clear hierarchical structure in an operation, which is also carried out in this way. But, apart from that, it is a lot of togetherness. […] You can be on first-name terms with the boss. […] It’s not that it always has to stay in a fixed structure after an operation. (Participant 3)

#### Theme 2: Leadership in the transition phase

The second central theme was leadership in the transition phase. The leader’s ability to give all followers the chance to voice their opinions and sensitivities was important, providing the opportunity to learn, reflect, and resolve inconsistencies. This sentiment was evidenced by the representative quotes below:

There isn’t any room for discussion on a mission! Not until the end of a mission. Then a leader can come and chat. […] Also, ask what did I experience and how I feel! Show interest; then I feel better. (Participant 2) After the mission, go and ask whether everything is fine and if there are any ideas. Ask if you can improve something. (Participant 4) To question leaders’ commands can be very negative on a mission! I get insecure. Then I question the executive. This is not working. If there is any dissatisfaction, it should be clarified in the follow-up. (Participant 9)

The leader’s behavior of listening to follower’s ideas and suggestions was also considered important (to enhance transparency and learning):

After a mission, it is also important to discuss. Experience must be shared. It is of no use if the wealth of experience that everyone has is kept to themselves. It’s useless. Exchange means learning through discussion. People listen to you. Should offer an open ear, be curious. (Participant 2) Transparent communication is very important. When you make a decision, you should also explain why it was necessary to do so afterward. Then, you can see a sense of your own security. Without justification and without explanation, you often don’t see any sense in it. (Participant 6)
